# Standardised snus packaging reduces brand differentiation: a web-based between-subject experiment

**DOI:** 10.1186/s12889-019-7763-4

**Published:** 2019-10-29

**Authors:** Torleif Halkjelsvik, Janne Scheffels

**Affiliations:** Department of Alcohol, Tobacco and Drugs, Norwegian Institute of Public Health, P.O. Box 222, Skoeyen, 0213 Oslo, Norway

**Keywords:** Plain packaging, Standardised packaging, Snus, Smokeless tobacco, Branding, Differentiation

## Abstract

**Background:**

Perceptions of tobacco packaging may be consequential for consumption and initiation. We explored the potential effect of standardised packaging on young adults’ ratings of the appeal of brands of snus (Swedish moist snuff) and on their perceptions of typical users of these brands. We were interested in both the effects on average levels of ratings and on the within-subject variability of the ratings. The latter was used as a measure of the extent to which individuals can differentiate between brands.

**Methods:**

A sample of 625 Norwegians aged 16–30 were randomly allocated to one of three between-subject conditions: Branded Packaging, Standardised Packaging, or Standardised Packaging with Health Warnings. The participants rated 10 snus brands on measures of general appeal and on their perceptions of the typical brand user (e.g., “… is sporty and active”).

**Results:**

The standardised packages (without health warnings) were not rated more negatively than the branded packages, while the standardised packages with health warnings were rated slightly more negatively than the branded packages. However, in terms of within-subject standard deviations, the variability of the brand ratings across the packages was substantially lower for standardised packaging types in comparison to branded packages.

**Conclusions:**

Even in cases where standardised tobacco packaging appears to have little overall effect on the valence of the average ratings, it can have a strong effect on the variability of the ratings. This suggests that standardised packaging can reduce the potential for brand differentiation.

## Background

Smoking has decreased dramatically in Norway in recent decades, while the use of snus (Swedish moist snuff) has increased [1]. The proportion of daily snus users is 12%, which is the same as the proportion of daily smokers [2]. During the period of increased snus use in Norway, a range of new snus brands and products have been introduced onto the market. A ban on all forms of tobacco advertising has meant that the main vehicle for advertising such new products has been their package design [3, 4]. The present study investigates whether removing this indirect form of advertisement by standardising the designs of snus packages will change the brands’ appeal and people’s perceptions of typical brand users.

Standardised packaging is assumed to contribute to reductions in the prevalence of tobacco use by reducing the attractiveness of tobacco products, eliminating the effects of tobacco packaging as a form of advertising and promotion, increasing the effectiveness of health warnings, and reducing the ability of retail packaging of tobacco products to mislead consumers about the harmful effects of smoking or using tobacco products [5].

Past experimental studies have concluded that standardised tobacco packages are perceived as less attractive than branded packages and that cigarettes in standardised packages are perceived to be of a lower quality [6]. Moreover, smokers of particular brands are viewed less favourably when the packages are standardised rather than branded, and some, but not all, studies have reported that health warnings are more salient and have a stronger impact when presented on standardised packages [6]. As examples of real life evidence of the impact of plain packaging, evaluation studies from Australia show that health warnings were noticed to a greater extent after standardised packaging was implemented; and that smokers experienced lower satisfaction, lower cigarette quality, and lower pack appeal than before the implementation [7]. Furthermore, Australian adolescents rated tobacco packages more negatively 1 year after the implementation of plain packages in comparison to before the implementation [8]. A recent Cochrane review summarizes the potential effects of standardised packaging more generally [9].

Based on the findings of previous studies, we expected that fully branded and standardized snus packages would differ in their overall appeal and their overall tendency to be associated with user characteristics. That is, we expected to observe mean differences in ratings between standardised packages and branded packages averaged over all brands and participants. In addition, we also expected that standardisation may affect the variability of ratings between different brands. If standardised packages were to reduce the variability of ratings, we would interpret this as an expression of a reduction of the potential to differentiate between brands.

A focal goal of marketing is to make a product different from other similar products in ways that matter to the preferences of buyers [10]. This process or state of product/brand differentiation can involve any aspect of the product, including packaging and features unrelated to the material product [11]. Product and packaging design is particularly important for differentiation in markets with similar products [12]. As one of several strategies to capture segments of the market, tobacco companies can position their products by means of user-brand associations [13]. A product positioned as “masculine” will attract different customers than a product associated with “sophisticated” users. In some cases, these marketing efforts may be highly successful, with the result that the general public becomes aware of the brand’s attributes (e.g., Marlboro). At other times, a brand’s appeal, or the perceptions of its typical users, may vary between social subgroups and between individuals. Interview studies of young tobacco users demonstrate that brands and package designs play a part in the construction of social identity, and that differentiation between brands is crucial for this process. The users experience that brands and package designs reflect their personal characteristics and act as markers of social status [14, 15].

Although much of the literature has focused on the appeal of standardised packages, standardised packaging can be defined simply as “packaging on which the surface graphics currently used to differentiate brands have been standardised,” [16] which does not refer to the attractiveness of the packages. There are a few studies that have explored standardised packaging and differentiation. Examples of these include studies that illustrate how average ratings vary between cigarette brands [16, 17], and studies that ask people directly whether packages are different from each other [8]. However, the present study is the first systematic attempt to analyse the variability of individual ratings. It is also the first to assess perceptions of standardised *snus* packaging in a country where snus use is highly prevalent (one study has been published from a U.S. context where the prevalence of snus use is low) [18].

The aim of the present study is to explore the potential differences between fully branded packages and two types of standardised packaging in terms of their average ratings on appeal and user-brand associations, and in terms of the variability within participants. We consider the latter to be an expression of the potential for brand differentiation.

## Method

### Participants

With the aim of having 200 participants in each experimental condition, an independent research agency invited 3400 members of their online panel, aged 16 to 30, to participate in the study. The panel consists of people recruited for the purpose of market research and social science surveys. The research agency aimed to provide a representative sample of the online population (i.e., people who use the internet), but younger people and people with no or short education are underrepresented in the panel. The sample size was determined on the basis of the effect size of a past study on cigarette packaging [19]. The online panel members were asked whether they consented to participate in research on the first page of the survey. Consent to participate was obtained from 672 persons, and 625 provided at least one rating of the snus packages (response rate = 18%). About 55% of the participants were female, and 22% were daily or weekly snus users. See Table 1 for further demographics. All measures, conditions, and data exclusions have been reported, except for the variables relating to aspects of snus use (e.g., years of snus use).

**Table 1:** Demographics of Net and Gross Samples

	Net Sample	Gross Sample
*N*	625	3400
Age		
Range	16–30	16–31
18>	12.6%	13.3%
18–22	30.1%	35.8%
22<	57.3%	50.9%
Gender		
Female	55.2%	44.0%
Snus Use		
Daily use	18.6%	–
Weekly use	3.3%	–
Tried/Occasional use	35.9%	–
Never used	42.3%	–
Education completed		
Elementary (10 year)	17.4%	–
College/Vocational	37.7%	–
Higher(< 4 years)	28.2%	–
Higher(> 4 years)	16.7%	–
Personal Income		
Below NOK 200000	40.3%	–
200′-299’	7.0%	–
300′-399	8.8%	–
400′-599’	17.2%	–
600′-799’	2.2%	–
> 799’	1.8%	–
Unwilling to report	22.6%	–

### Procedure

The pictures of snus packages and the questionnaire was presented as an online form in a web browser and filled out by the participants at home or in any other place of choice. The participants were randomly assigned to one of three experimental conditions. One group (*n* = 215) rated 10 original snus packages (Branded condition), another group (*n* = 218) rated the same 10 brand names presented as standardised packages without health warning labels (Standardised non-HWL), and the last group (*n* = 192) rated the same 10 brands presented as standardised packages with health warning labels (Standardised HWL). We selected brands that reflected different package designs among the most sold brands, but excluded brands that were of similar design (e.g., variations of the same general brand). The brands were presented in random order.

### Materials and measures

We tested two types of standardised designs—one design without health warnings on the front of the packages (which is the current standardised packaging that was implemented in Norway in July 2018), and one design with a text-only health warning on the front of the packages (which is likely the future design when Directive 2014/40/EU is implemented in Norway). The experiment was carried out in October 2015—well in advance of the implementation of standardised packaging.

Photos of the original snus packages were obtained from tobacco industry web pages. On the basis of a model provided by the Norwegian Directorate of Health, a digital design studio developed realistic pictures of 10 standardised packages and 10 standardised packages with health warning labels.

The appeal of the packages was measured with 7-point sliders anchored by the word pairs “Unattractive-Attractive”, “Repulsive-Appealing”, and “Do not want to try/use this-Want to try/use this”, items that were partly based on past research [19–21].

Six user-brand associations were derived from narratives in focus group studies on cigarette packaging [14, 22]. The participants received the statement “I picture the typical user...” for each of the 10 snus variants, and they rated the following items on 6-point sliders from “Does not fit well” to “Fits well”: “...is social and has many friends”; “...is urban and hip”; “...is sporty and active”; “...is elegant and stylish”; “...wants to be unique/have her[/his] own style”; “...is rough/tough”. Altogether, participants made 90 ratings of the snus brands (10 snus variants, 3 appeal items, and 6 user-association items).

### Statistical analyses

Confidence intervals for differences between the conditions were obtained from t-tests and repeated ANOVAs in SPSS 24. For the analyses of appeal, the three appeal items were first aggregated for each brand rating, and the means and standard deviations of these scores were then calculated across the brands for each participant. The analyses of the user-brand associations were based on each individual’s mean ratings across the ten brands and each individual’s standard deviation of the ten ratings.

## Results

### Mean differences in appeal

Table 2 presents the average ratings for the three conditions for all the outcome measures. The average ratings were in the range 3.5 to 3.7 which is somewhat below 4, the midpoint of the scale (i.e., ratings were slightly negative). When considering the differences between the Branded condition and the Standardised non-HWL, there appears to be no effect of the conditions on the general ratings of appeal, the difference (Branded minus Standardised non-HWL) was − 0.01, CI^95%^[− 0.38, 0.29].

**Table 2:** Average Snus Brand Ratings (Standard Deviations in Parenthesis) and Average Within-Participant Variability

	General Appeal	User-Brand Associations					
		Social	Urban	Sporty	Elegant	Unique	Tough
Mean Ratings							
Branded	3.75 (1.35)	3.34 (1.17)	3.02 (1.17)	2.68 (1.09)	2.67 (1.16)	2.96 (1.19)	3.06 (1.17)
Standardised non-HWL	3.76 (1.60)	3.07 (1.16)	2.82 (1.18)	2.44 (1.11)	2.54 (1.19)	2.75 (1.23)	2.99 (1.28)
Standardised HWL	3.54 (1.60)	3.06 (1.24)	2.83 (1.16)	2.32 (1.19)	2.40 (1.14)	2.68 (1.16)	3.05 (1.29)
Within-Participant Variability							
Branded	1.17	0.85	0.96	0.92	0.95	1.09	1.21
Standardised non-HWL	0.91	0.79	0.88	0.80	0.84	0.92	0.99
Standardised HWL	0.86	0.78	0.88	0.63	0.73	0.87	0.99

Number of participants: Branded = 191–215, Standardised non-HWL = 180–218, Standardised HWL = 168–192. Within-participant variability is the average of each participants’ standard deviation of ratings over the different brands

In the case of the comparison Branded versus Standardised HWL, there was a tendency in the expected direction for the appeal measure, Difference = 0.21, CI^95%^[− 0.08, 0.50], which corresponds to about a 6% lower appeal rating in the Standardized HWL condition (see Table 2).

### Mean differences in user-brand associations

In terms of user-brand associations, the difference between the Branded and the Standardised non-HWL conditions across the six social dimensions was 0.21, CI^95%^[0.01, 0.41], which corresponds to about a 7% lower endorsement of user characteristics in the Standardised non-HWL. In separate analyses of the six dimensions, the differences ranged from 0.26, CI^95%^[0.04, 0.49] on the dimension of Social to 0.08, CI^95%^[− 0.16, 0.31], on the dimension of Tough.

The difference between the Branded and the Standardised HWL conditions across the six dimensions was 0.23, CI^95%^[0.03, 0.44], which corresponds to lower ratings of about 8% in the Standardised HWL condition. When analysed separately for each user-brand dimension, the differences ranged from 0.28, CI^95%^[0.04, 0.51] on the dimension of Social to 0.01, CI^95%^[− 0.23, 0.26], on the dimension of Tough.

The analyses on mean differences between the branded packaging and the two types of standardised packaging revealed no, or only small, effects in terms of the overall appeal and in terms of the user-brand associations. As suggested in the introduction, there may also be difference between the conditions in terms of the variability of ratings. This is addressed in the analyses of the differences in intra-individual variability presented below. The variability is operationalized as the participants’ standard deviations of their ratings of the ten brands.

### Within-participant variability of appeal ratings

The difference in average within-subject variability of appeal ratings between Branded and Standardised non-HWL packaging was 0.27, CI^95%^[0.13, 0.41], corresponding to a reduction of the average standard deviation of about 23% from branded to standardised. That is, the typical spread (standard deviation) of ratings among the participants when rating fully branded packages was ± 1.17 points, whereas the typical spread in the Standardised non-HWL was ± 0.91 points (see Table 2).

For the Branded versus Standardised HWL comparison, the difference in average variability of appeal was 0.31, CI^95%^[0.16, 0.47], corresponding to a reduction of about 26% from branded to standardised.

### Within-participant variability of user-brand ratings

In terms of the within-subject variability of user-brand associations, the difference between Branded and Standardised non-HWL packaging across the six dimensions was 0.14, CI^95%^[0.04, 0.24], which corresponds to a reduction of the average standard deviation of about 14% from branded to standardised. With regard to the six dimensions separately, the difference ranged from .06, CI^95%^[− 0.04, 0.16], on the dimension of Social to 0.22, CI^95%^[0.08, 0.35], on the dimension of Tough.

The difference between Branded and Standardised HWL across the six dimensions was 0.20 CI^95%^[0.10, 0.30], which corresponds to a 20% reduction of the average standard deviation. The differences ranged from .07, CI^95%^[− 0.04, 0.17], on the dimension of Social to 0.29, CI^95%^[0.16, 0.41], on the dimension of Sporty.

Thus, the analyses of variability demonstrated substantial reductions in the participants’ standard deviations of ratings for the standardised conditions in comparison with the fully branded condition. In addition to the above analyses, a similar pattern of results was found in Linear Mixed Models that used the raw, unaggregated data (see Additional file 1). There was no moderation by gender or by snus use status (daily users versus others).

## Discussion

The analyses did not detect differences in the mean ratings of general appeal between the fully branded packages and the standardised packages without health warnings. There were only small differences in the general appeal ratings between the branded packages and the standardised packages with health warnings, and small differences in the endorsements of user-brand associations, with effects corresponding to about 7–8% lower mean ratings in the standardised conditions. By contrast, the analyses of the intra-individual variability of the brand appeal ratings revealed clear differences between the conditions, which corresponded to about 25% lower standard deviations for the two standardised packaging conditions in comparison to the branded condition. A clear, but somewhat weaker, effect was found for the overall differences in the variability of user-brand associations. Outcome measures that in the analyses of the average ratings appeared to be unaffected by packaging types could display substantial differences in the analyses of the variability. For instance, the difference in mean ratings between the branded and the standardised packaging types for the dimension of Tough was practically zero, but the average within-participant standard deviation was about 18% lower for both types of standardised packages in comparison to the branded packaging.

In contrast to the majority of past studies on standardised tobacco packaging [6], we found only small average differences between the branded and the standardised packages, and no difference when comparing the appeal ratings of the fully branded packaging with the ratings of the standardised packages without warnings. In the literature on standardised cigarette packaging, the strongest effects appear to be documented in studies that elicit direct comparisons between standardised and branded cigarette packages [23, 24]. However, past studies have also found higher ratings of appeal for branded packages in comparison to standardised packages in between-subject designs [20, 21, 25]. One difference between the current study and most of the previous literature is the extent of stimuli sampling. Two or three products [20, 25], and sometimes only one [23, 26], has typically been selected for comparison with standardised versions of the same product, which may have made the results vulnerable to the researcher’s choice of stimuli [27, 28].

The studies that have used a larger set of stimuli have typically employed outcome measures based on the participants’ number of positive ratings (coding the negative responses in the same category as the neutral responses) [21, 29–31]. The more positive ratings of branded packages in these studies could reflect both a shift in the valence of ratings and more variation in the ratings (i.e., more positive *and* more negative). In an attempt to investigate whether the coding of responses could explain the contradictory results, we performed a reanalysis of a Norwegian study on standardised cigarette packaging (see Additional file 1). The cigarette packaging study originally used the number of positive ratings as the outcome measure, and reported the typical result in the literature; on average, the branded packages were rated more positively than the standardised packages in terms of appeal, and received higher ratings on positive user-brand associations [21, 32].The reanalysis indicated that there were indeed more positive ratings for a branded cigarette packaging condition in comparison to a standardised cigarette packaging condition, but not an overall shift in the mean positivity of the scores. That is, there were both more positive ratings *and* more negative ratings of branded packages, which is consistent with the present findings of a larger spread of ratings for branded packages in comparison to standardised packages.

Taken together with the reanalysis of the study on cigarette packaging, the present results suggest that standardised packaging may be an effective strategy for combatting tobacco use by reducing the potential for brand differentiation. When the graphical design elements are removed, people are less able to associate packages with certain personal and social characteristics of product users, and they are less able to find brands that are personally appealing. While the standardised packages still had their brand names and variant descriptors, the present study indicates that such information is insufficient for maintaining differentiation to the same extent as fully branded packages. Although the study was not designed to test differences between standardised packaging with and without HWLs, the results point in the direction that standardised packaging *with* HWLs may be more effective in lowering the attractiveness of snus.

One threat to the generalisability of the results is the fact that the sample consisted of relatively young Norwegian members of an online research panel. However, the recruitment of youth can be justified by the fact that young people are the policy’s main target group [33]. Another limitation is that the study’s setting was highly artificial. The participants only viewed pictures of standardised packages, possibly for the very first time, and did not experience the actual products.

We were able to replicate the main results in a reanalysis of data from a previously published study on cigarette packaging. This means that it is unlikely that the results were due to perceptions that are unique to snus. The fact that the two studies used different research firms for data collection, together with different response scales and different wording of the questions, further strengthens our findings.

We chose to report the combined results for both snus users and non-users. The Linear Mixed Models (see Additional file 1) revealed no interaction between condition and snus use status, and the pattern of results for variability was fairly similar for non-users and snus users, although the effects for the latter group were somewhat less consistent (possibly due to a smaller sample size).

## Conclusion

Standardised packaging is associated with less variability of brand ratings, which suggests that it reduces people’s ability to differentiate between brands. In keeping with the original idea of plain packaging [16], removing package designs makes it more difficult for people to find brands that are personally appealing. Likewise, it makes it more difficult for tobacco companies to build brands that exploit people’s desires to identify with certain social groups or personal characteristics.

## Supplementary information

**Additional file 1.** Standardised Tobacco Packaging Reduces Brand Differentiation: a Web-Based Between-Subject Experiment. Supplemental analyses of the data and a reanalysis of a similar dataset on cigarette packaging.

## Data Availability

The datasets used and/or analysed during the current study are available from the corresponding author on reasonable request.

## Abbreviation

HWL: Health Warning Labels
