# Supplementary material for: Standardised snus packaging reduces brand differentiation: a web-based between-subject experiment
Source: BMC Public Health. 2019 Oct 29;19:1414. doi: 10.1186/s12889-019-7763-4 (PMC6821023; doi:10.1186/s12889-019-7763-4)
Supplement: Supplementary file 1 — Additional file 1. Standardised Tobacco Packaging Reduces Brand Differentiation: a Web-Based Between-Subject Experiment. Supplemental analyses of the data and a reanalysis of a similar dataset on cigarette packaging. [file 12889_2019_7763_MOESM1_ESM.docx]

**Additional file 1**

**for**

**"Standardised Snus Packaging Reduces Brand Differentiation: a Web-Based Between-Subject Experiment"**

**Torleif Halkjelsvik & Janne Scheffels**

**Norwegian Institute of Public Health**

**LINEAR MIXED MODELS**

**Methods, Linear Mixed Models**

As a complement to the simple analyses of aggregated ratings in the article, we performed a series of Linear Mixed Model (Multi-Level Model) analyses on the raw, unaggregated data. The analyses were carried out in the software R (R Core Team, 2015), with packages “lme4” (Bates, Maechler, Bolder, & Walker, 2014) and “lmerTest” (Kuznetsova, Brockhoff, & Christensen, 2015), and in STATA 14.1 (with the command "xtmixed"). In these analyses we also report Bayes Factors for differences in mean ratings. Bayes Factors were obtained from an online calculator (Dienes, 2011), and priors were based on effect sizes calculated from the results on "positive pack characteristics" (Appeal) and "positive smoker characteristics" (User-Brand Associations) from Wakefield et al. (2012). For further details, see section on Priors.

To allow for different degrees of variability between branded and standardised packages, we estimated the standard deviations of random effects separately for each experimental condition. This was done by means of *variance functions*. More specifically, we generated dummy variables, one for each of the three experimental conditions, and used these to estimate random slopes for each of the conditions without any intercepts of random effects (see Rabe-Hesketh & Skrondal, 2012, p.362). We did this for each experimental condition for snus users and non-users, respectively, giving 6 different standard deviations for each random effect.

We estimated three sources of variability, the variability of Participants (between-participant variability; which was not of central concern in the present analyses), the variability of Brands (between-brands variability aggregated over participants), and the variability of the Participant by Brand interaction (which gives the within-participant variability after accounting for the variability of average brand ratings). When the latter was inestimable due to only one item per measure, we used the residuals as proxies for the within-person variability, and estimated separate residual standard deviations for each condition in each user status group (option "residuals" in STATA).

As tests of differences in standard deviations for random effects and for residuals, we performed Likelihood-Ratio Tests (LRTs) where models with separate standard deviations for Standardised and Branded packaging were compared with models with standard deviations constrained to be equal across conditions (see Snijders & Bosker, 2012, pp.96-97).

**Results, Linear Mixed Models**

*Mean Differences in Appeal*

Table S1 presents the results of the model with appeal ratings as the dependent measure. The fixed effects of Condition presented in the first rows of Table S1 show that Standardised non-HWL packaging and original Branded packaging achieved approximately similar ratings of appeal, Difference = -0.08, CI^95%^[-0.34, 0.17], whereas Standardised HWL were rated 0.26 points lower than Branded Packages on the 7-point scale, CI^95%^[-0.52, 0.00]. In other words, we were unable to document that standardised packages per se (i.e., non-HWL) could reduce the appeal of snus. Still, there was a tendency of Standardised HWL to be rated somewhat lower than original packages. The main effects of the covariates Rating Type (differences between the three appeal items) and Snus Use Status were not of interest in the present study.

*Table S1. Linear Mixed Model of Appeal Ratings for Snus Brands.*

|  |  |  | | | Regression  Coefficient | | Standard Error |
| --- | --- | --- | --- | --- | --- | --- | --- |
| *Fixed effects* | Intercept |  | | | 3.78 | | 0.092 |
|  | **Condition** | Branded | | | Ref. | |  |
|  |  | Standardised | | | -0.08 | | 0.130 |
|  |  | Standardised w/ Warning | | | -0.26 | | 0.133 |
|  | **Rating Type** | Attractive | | | Ref. | |  |
|  |  | Appealing | | | -0.13 | | 0.023 |
|  |  | Want to try | | | 0.00 | | 0.023 |
|  | **Snus Use Status** | Non-user | | | Ref. | |  |
|  |  | User | | | 0.44 | | 0.111 |
|  |  |  | | |  | |  |
| *Random effects* |  |  | | | Standard Deviation | | |
|  | **Participants** | Non-users | | | |  | |
|  |  |  | Original | | | 1.25 | |
|  |  |  | Standardised | | | 1.62 | |
|  |  |  | Standardised w/warning | | | 1.69 | |
|  |  | Users | | | |  | |
|  |  |  | Original | | | 0.77 | |
|  |  |  | Standardised | | | 0.94 | |
|  |  |  | Standardised w/warning | | | 0.89 | |
|  | **Participants by Brands** | Non-users | | | |  | |
|  |  |  | | Original | | 0.92 | |
|  |  |  | | Standardised | | 0.64 | |
|  |  |  | | Standardised w/warning | | 0.69 | |
|  |  | Users | | | |  | |
|  |  |  | Original | | | 1.39 | |
|  |  |  | Standardised | | | 1.20 | |
|  |  |  | Standardised w/warning | | | 1.00 | |
|  | **Residuals** |  | | | | 1.08 | |

*Note.* "Ref." = reference category coded 0. Number of participants in each condition: Non-Users= 142-170, Users = 45-50. Participants by Brands in each condition: Non-users = 1134-1363, Users = 378-393. Residuals = 13645.

To interpret the non-significant Branded versus Standardised non-HWL comparison, we calculated Bayes Factor (Dienes, 2011). As the *prior* we used a half-normal distribution with mean 0 and a standard deviation of 0.7 (based on the effect size in Wakefield, et al. 2012). This yielded a Bayes Factor (BF) of 0.32, meaning that, given this expectation, the null hypothesis is three times more likely than the alternative hypothesis. In comparison, the effect of Branded versus Standardised HWL yielded a BF of 2.34, suggesting more support for the presence than the absence of an effect, but still indicating insensitive data.

Exploratory analyses revealed no substantial interaction between snus use status and Conditions, *F* = 0.28, no effect of gender, *t* = 0.93, or the interaction gender by Conditions, *F* = 1.32, and no effect of age, *t* = 0.87, or the interaction age by Conditions, *F* = 0.05.

*Variability of Appeal*

The effect representing how brands differ from each other in terms of average ratings would normally have been represented by a random effect of Brands (variation in average brand ratings between the 10 different brands), but this term was omitted from the model because the variance was estimated as zero. This means that, on average, no packages stood out either positively or negatively.

The measure of within-participant variability was represented by the Participants by Brands interaction in Table S1. For non-users of snus, the Participants by Brands standard deviations of the two standardised packaging types were about 25-30% lower than the standard deviation of the original Branded packaging, χ^2^ (*df* = 1) > 30, *p*s < .001. There was no substantial difference between the two types of standardised packages, χ^2^ (*df* = 1) = 1.45, *p* = .22.

For snus users, the standard deviations of Standardised non-HWL and Standardised HWL were 14% and 28% lower than the standard deviation of Branded packaging, χ^2^ (*df* = 1) > 4.26, *p*s < .05 for tests of all pairwise differences. In other words, the data showed a clear effect of standardised packaging for both users and non-users, with less variability of standardised package types compared with the original branded designs.

In summary, the analyses for the outcome Appeal showed that there was no difference between standardised non-HWL snus packages and branded snus packages in terms of the overall level of ratings, but a tendency of less favourable ratings for standardised HWL in comparison with branded packages. Nevertheless, there were robust reductions in the variability of brand ratings for both types of standardised packaging in comparison with branded packaging. These analyses largely confirmed the results of the simpler analyses reported in the article.

*Mean Differences* *of* *User-Brand Associations*

Table S2 presents results for separate analyses of each of the 6 user-brand associations (a full model was not able to converge). Concerning mean differences between conditions, the comparisons between Branded and Standardised non-HWL only gave one *p*-value below .05, for the user-brand association Social, reflecting slightly lower endorsement in the Standardised condition than in the Branded condition (0.25 points on the 6-point scale; Bayes Factor = 3.55). The results for Urban, Sporty, Elegant, Unique, and Tough were inconclusive (differences between 0 and 0.2 points; Bayes Factors from 0.5 to 1.5).

The comparisons between Branded and Standardised HWL gave *p*-values below .05 for the dimensions Social, Sporty, Elegant and Unique, reflecting ratings about 0.3 points lower for the Standardised with Warning condition (BFs from 3.82 to 8.18). For Urban and Tough the results were inconclusive (differences below 0.2; Bayes Factors 1.35 and 0.44).

These results were largely consistent with the conclusions in the main article, but also suggest that the effect found for mean differences in user-brand associations between the Branded and Standardized non-HWL may not be reliable.

*Table S2. Unstandardised Regression Coefficients (B) of Fixed Effects and Standard Deviations of Random Effects (SD) from Linear Mixed Models of User-Brand Associations for Snus Brands.*

|  | | | | | | |  | | **Social** | **Urban** | **Sporty** | **Elegant** | **Unique** | **Tough** |
| --- | --- | --- | --- | --- | --- | --- | --- | --- | --- | --- | --- | --- | --- | --- |
| *Fixed effects* | | | | | | |  | | *B* | *B* | *B* | *B* | *B* | *B* |
|  | Intercept | | | | | |  | | 3.26 | 2.92 | 2.46 | 2.56 | 2.87 | 2.88 |
|  | **Condition** | | | | | | Branded | | ref. | ref. | ref. | ref. | ref. | ref. |
|  |  | | | | | | Standardised | | -0.25^*^ | -0.19 | -0.15 | -0.11 | -0.21 | -0.02 |
|  |  | | | | | | Standard.w/warning | | -0.27^*^ | -0.19 | -0.32^*^ | -0.28^*^ | -0.29^*^ | 0.04 |
|  | **Snus Use** | | | | | | Non-user | | ref. | ref. | ref. | ref. | ref. | ref. |
|  |  | | | | | | User | | 0.36^**^ | 0.45^**^ | 0.64^**^ | 0.46^**^ | 0.42^**^ | 0.38^**^ |
| *Random effects* | | | | | | | |  | *SD* | *SD* | *SD* | *SD* | *SD* | *SD* |
|  | | **Participants** | | | | | | |  |  |  |  |  |  |
|  | |  | Non-Users | | | | | |  |  |  |  |  |  |
|  | |  |  | | Branded | | | | 1.16_a_ | 1.09_a_ | 1.10_a_ | 1.09_a_ | 1.06_a_ | 1.06_a_ |
|  | |  |  | | Standardised | | | | 1.05_a_ | 1.08_a_ | 0.96_a_ | 1.04_a_ | 1.07_a_ | 1.16_a_ |
|  | |  |  | | Standardised w/warning | | | | 1.08_a_ | 0.98_a_ | 0.97_a_ | 0.98_a_ | 1.02_a_ | 1.11_a_ |
|  | |  | Users | | | | | |  |  |  |  |  |  |
|  | |  |  | | Branded | | | | 0.66_a_ | 0.93_a_ | 0.63_a_ | 0.82_a_ | 0.89_a_ | 0.72_a_ |
|  | |  |  | | Standardised | | | | 1.11_b_ | 0.96_a_ | 1.07_b_ | 1.07_a_ | 1.08_a_ | 0.98_ab_ |
|  | |  |  | | Standardised w/warning | | | | 1.26_b_ | 1.15_a_ | 1.27_b_ | 1.16_a_ | 1.05_a_ | 1.24_b_ |
|  | | **Brands** | | | | | | |  |  |  |  |  |  |
|  | |  | Non-Users | | | | | |  |  |  |  |  |  |
|  | |  |  | | Branded | | | | 0.24_a_ | 0.34_a_ | 0.33_a_ | 0.22_a_ | 0.43_a_ | 0.68_a_ |
|  | |  |  | | Standardised | | | | 0.13_a_ | 0.15_b_ | 0.14_b_ | 0.15_a_ | 0.25_b_ | 0.36_b_ |
|  | |  |  | | Standardised w/warning | | | | 0.16_a_ | 0.17_b_ | 0.10_b_ | 0.18_a_ | 0.14_b_ | 0.44_b_ |
|  | |  | Users | | | | | |  |  |  |  |  |  |
|  | |  |  | | Branded | | | | 0.21_a_ | 0.30_a_ | 0.25_a_ | 0.21_a_ | 0.14_a_ | 0.84_a_ |
|  | |  |  | | Standardised | | | | 0.23_a_ | 0.34_a_ | 0.36_a_ | 0.31_a_ | 0.23_a_ | 0.46_a_ |
|  | |  |  | | Standardised w/warning | | | | 0.08_a_ | 0.20_a_ | 0.07_b_ | 0.21_a_ | 0.05_a_ | 0.43_a_ |
|  | | **Residuals** | | | | | | |  |  |  |  |  |  |
|  | |  | | Non-Users | | | | |  |  |  |  |  |  |
|  | |  | |  | | Branded | | | 0.98_a_ | 1.05_a_ | 0.99_a_ | 1.09_a_ | 1.15_a_ | 1.17_a_ |
|  | |  | |  | | Standardised | | | 0.92_b_ | 0.98_b_ | 0.92_b_ | 1.04_a_ | 1.09_a_ | 1.12_a_ |
|  | |  | |  | | Standardised w/warning | | | 0.90_b_ | 0.99_b_ | 0.75_c_ | 0.86_b_ | 1.02_b_ | 1.05_b_ |
|  | |  | | Users | | | | |  |  |  |  |  |  |
|  | |  | |  | | Branded | | | 0.94_a_ | 1.17_a_ | 1.16_a_ | 1.14_a_ | 1.22_a_ | 1.16_a_ |
|  | |  | |  | | Standardised | | | 0.87_a_ | 1.06_ab_ | 0.91_b_ | 0.92_b_ | 1.03_b_ | 1.15_a_ |
|  | |  | |  | | Standardised w/warning | | | 0.92_a_ | 1.03_b_ | 0.89_b_ | 0.92_b_ | 1.10_ab_ | 1.12_a_ |

*Note.* * *p* < .05, ** *p* < .01 for fixed effects. ref. = reference category coded 0. Subscripts a, b, and c: within each user group (Non-users and Users), no shared subscript means *p* < .05 for differences between random effects. Participants N = 583-622, Brands N = 10, Residuals N =4405-4998.

*Variability of User-brand Associations*

For non-users of snus, the random effect of Brands suggested reduced variability in the brands' average ratings (aggregated over participants) for both types of standardised packaging for most of the user-brand associations, with reductions in standard deviations ranging from 18% to 70% (average reduction 46%). Among snus users, there was no robust or systematic decrease in the variability of average user-brand ratings.

In Table S2, the within-participant variability is represented by the Residuals (because ratings were made only once per dimension per brand). For non-users of snus, there was a clear pattern of reduced variability in the two standardised packaging conditions (decreases in standard deviations ranging from 4% to 24%, on average 10 %). The pattern was less consistent for snus users, although the variability was reduced for some of the dimensions.

In summary, the results for the outcome measures representing user-brand associations (Social, Urban, Sporty, Elegant, Unique, and Tough) showed weak to no effects of standardised packaging per se in terms of mean differences. That is, standardised packages without health warning did not consistently reduce positive user associations in comparison with branded packages (inconclusive data), but standardised packages with health warnings reduced positive user associations compared to branded packages on 4 out of 6 outcomes. As for the analyses of variability, snus non-users showed a clear pattern of reduced variability of brand ratings when packages where standardised. Snus users showed a less consistent pattern, perhaps due to smaller sample size.

**Priors for Bayes Factors**

The priors for calculation of Bayes Factor were based results from Wakefield et al. (2012). The method of the study by Wakefield et al. (2012) differs from the present one in several respects (e.g., different number of measurements and no random factors in the analyses), still, we considered the methods as fairly similar, similar enough to rely on the results as a rough indication of the expected effect sizes. In Wakefield et al. (2012), the mean difference between standardised and branded packages for the measure "positive pack characteristics" was about 0.45 standard deviations, as we could calculate from the F-statistics and the number of participants.

Our Participants random effect (See Table S1), would roughly correspond to the standard deviations when aggregating over items and brands (which was done by Wakefield et al., 2012), and across conditions the standard deviation of the Participants effect was approximately 1.5. This means that we expected that standardised packages would be rated about 0.7 lower than branded packages on a 7-point brand appeal scale (0.45*1.5 = 0.7). We followed the recommendations by Dienes (2014) and set the prior to a half-normal distribution of effect sizes with mean 0 and standard deviation 0.7.

The effect size for the "positive smoker characteristics" effect in Wakefield et al. (2012) was 0.36, which for our user-brand association measures would correspond to about a 0.4-point difference on the 6-point scale (assuming an SD of about 1.1). For simplicity, we used 0.4 points as the standard deviation of the prior for all user characteristics, despite differences in standard deviations between the different characteristics.

**REANALYSIS OF CIGARETTE PACKAGING DATA**

**Method, Reanalysis**

Although the cigarette packaging dataset we reanalysed has been thoroughly described in previous publications (Lund & Scheffels, 2013; Scheffels & Lund, 2013), a quick overview of the sample, materials and design follows. Participants were randomly assigned to a Branded condition, a Standardised condition, or a condition with standardised packaging with no descriptors of the brand variant. However, we only analysed the two first conditions, as these were most similar to our study on snus packaging. This sample consisted of 695 participants aged 15 to 22 (60% female), of whom 143 were smokers. The data were collected before the Norwegian authorities proposed Pantone 448C as the colour of standardised tobacco packages, therefore, the standardised packages were grey.

Each participant rated 12 of 20 different brands. The question measuring product appeal asked: "In comparison with other brands, how appealing is this cigarette brand?", with response options "Less attractive", "No difference", "More attractive", and "Don't know".

**Results and Discussion, Reanalysis**

When positive ratings were coded 1, negative -1, and “No difference”/”Don’t know” 0, the average appeal score was -0.01 for the Branded condition and 0.00 for the Standardised condition, difference = -0.01, CI^95%^[-0.05, 0.02]. Apparently, this suggests that there were no effects of standardised packaging on appeal ratings. However, Table S3 shows that there is a larger spread of ratings for the Branded condition, as there are more positive *and* more negative ratings. Compared to Standardised packages, the average proportion of positive ratings for the Branded condition was 0.06 points higher, CI^95%^[0.04, 0.09], and the average proportion of negative ratings was 0.08 points higher, CI^95%^ [0.05, 0.11]. Thus, there were more positive ratings in the Branded condition, but this was due to the spread of ratings, not due to an overall shift in the positivity of the scores.

*Table S3. Average Proportions of Positive and Negative Brand Ratings, Reanalysis of Cigarette Packaging Data.*

|  |  | Positive ratings | | Negative ratings | |
| --- | --- | --- | --- | --- | --- |
|  | N | M | SD | M | SD |
| Branded | 382 | 0.25 | 0.19 | 0.26 | 0.19 |
| Standardised | 313 | 0.18 | 0.18 | 0.18 | 0.19 |

**REFERENCES**

Bates, D., Maechler, M., Bolder, B., & Walker, S. (2014). lme4: Linear mixed-effects models using Eigen and S4. R package version 1.1-7, http://CRAN.R-project.org/package=lme4.

Dienes, Z. (2014). Using Bayes to get the most out of non-significant results. *Frontiers in Psycholology, 5*: 781. doi: 10.3389/fpsyg.2014.00781

Kuznetsova, A., Brockhoff, P. B., & Christensen, R. H. B. (2015). lmerTest: Tests in Linear Mixed Effects Models. R package version 2.0-29. http://CRAN.R-project.org/package=lmerTest.

Lund, I., & Scheffels, J. (2013). Young smokers and non-smokers perceptions of typical users of plain vs. branded cigarette packs: a between-subjects experimental survey. *BMC Public Health, 13*(1):1005

R Core Team. (2015). R: A language and environment for statistical computing. R Foundation for Statistical Computing, Vienna, Austria. https://www.R-project.org.

Rabe-Hesketh, S., Skrondal, A. & Pickles, A. (2004). *GLLAMM Manual.* U.C. Berkeley Division of Biostatistics Working Paper Series, Paper 160. University of California, Berkeley

Scheffels, J., & Lund, I. (2013). The impact of cigarette branding and plain packaging on perceptions of product appeal and risk among young adults in Norway: A between-subjects experimental survey. *BMJ Open,* 3(12).

Snijders, T. A. B., & Bosker, R. J. (2012). *Multilevel Analysis: An Introduction to Basic and Advanced Multilevel Modeling, second edition.* . London: Sage Publishers.

Wakefield, M., Germain, D., Durkin, S., Hammond, D., Goldberg, M., & Borland, R. (2012). Do larger pictorial health warnings diminish the need for plain packaging of cigarettes? Addiction, 107(6), 1159-1167
